# Beneficial effect modification on survival outcome of sepsis between ART-123 and polymyxin B‑immobilised haemoperfusion: a nationwide Japanese registry study

**DOI:** 10.1186/s13613-020-00674-8

**Published:** 2020-05-13

**Authors:** Katsunori Mochizuki, Kotaro Mori, Hiroshi Kamijo, Michitaro Ichikawa, Kenichi Nitta, Hiroshi Imamura

**Affiliations:** Department of Emergency and Critical Care Medicine, Shinshu University School of Medicine, 3-1-1 Asahi, Matsumoto, Nagano 390-8621 Japan

**Keywords:** Sepsis, Thrombomodulin, ART-123, Polymyxin B‑immobilised haemoperfusion, Effect modification

## Abstract

**Background:**

Although recently published randomised controlled trials did not confirm significant positive effect of ART-123 or polymyxin B‑immobilised haemoperfusion (PMX-HP) on survival outcome, previous studies using a dataset of 3195 patients with sepsis registered at 42 intensive care units throughout Japan revealed significantly reduced mortality following these treatments. A study has suggested the efficacy of combination therapy with ART-123 and PMX-HP; however, it did not evaluate the effect modification between them. We hypothesised that coadministration of ART-123 and PMX-HP has a significant positive effect modification on survival outcome. The purpose of this study was to evaluate the effect modification between ART-123 and PMX-HP treatment on the survival outcome of sepsis using post hoc analysis of the dataset of the Japan Septic Disseminated Intravascular Coagulation registry.

**Results:**

Of the 3195 patients recorded in the registry, 2350 were analysed. The product term between ART-123 and PMX-HP was analysed by the Cox regression model to evaluate significance. The primary outcome of this study was hospital mortality. Although the administration of ART-123 was independently positively associated with survival outcome (adjusted hazard ratio [HR]: 0.834, 95% confidence interval [CI] 0.695–0.999; *P* = 0.049) in the model prior to the introduction of the product term, a significant effect modification on survival outcome was observed between the administration of ART-123 and PMX-HP treatment (adjusted HR: 0.667, 95% CI 0.462–0.961; *P* = 0.030).

**Conclusions:**

The main effect of the administration of ART-123 may be beneficial for survival outcome in patients with sepsis. In addition, a significant beneficial effect modification on survival outcome was observed between the administration of ART-123 and PMX-HP treatment.

## Background

The survival outcome of sepsis is improving with the adoption of standards for treatment, such as the Surviving Sepsis Campaign Guidelines [1–4]. However, mortality is still high, and sepsis, a complicated condition characterised by life-threatening organ dysfunction secondary to infections, remains an important worldwide public health issue [5–7]. Along with the core treatment approaches for infections (antibiotic therapy and source control), various additional treatments to control pathophysiological pathways leading to organ dysfunction have been investigated, with the goal of reducing the morbidity and the mortality of sepsis [6, 8].

The coagulation pathway, which mediates coagulopathy and disseminated intravascular coagulation (DIC) in sepsis, has been one such research target. Coagulopathy is a complication of sepsis that causes organ dysfunction and leads to high mortality [9–13]. In addition, coagulopathy and sepsis adversely affect each other via crosstalk between coagulation and inflammation pathways [14]. Therefore, several anticoagulants expected to control coagulopathy and reduce the mortality of sepsis have been investigated [15–17]. ART-123 (recombinant human soluble thrombomodulin) is a novel anticoagulant that also has an anti-inflammatory effect [18]. Although the clinical efficacy of ART-123 in reducing the mortality of sepsis has been thoroughly investigated [19–24], its effects were not significant in a recently published phase 3 randomised controlled trial (RCT) known as the SCARLET trial [25].

Activation of the endotoxin pathway induces organ dysfunction and shock in patients with Gram-negative microorganism infections [26]. To control the systemic inflammatory response, endotoxin removal using polymyxin B‑immobilised haemoperfusion (PMX-HP) has been attempted [27, 28]. PMX-HP therapy was expected not only to stabilise the shock response in the hyperinflammatory phase, but also to alleviate the subsequent immunosuppressive phase, known as immunoparalysis, which causes secondary infections and increased mortality [29, 30]. However, although the clinical efficacy of the PMX-HP therapy in improving the survival outcome was initially expected [31], no significant mortality reduction was observed in larger, more recent RCTs [32, 33], as well as in the meta-analysis that included those RCTs [34].

Although recently published RCTs have not confirmed the significant positive effects of ART-123 and PMX-HP on sepsis survival outcomes, previous studies using a dataset of 3195 registered adult patients with sepsis revealed significant efficacy for these approaches in reducing hospital mortality [35, 36]. It has been suggested that specific target populations may obtain survival benefits from these therapies [24, 37, 38]. Therefore, differences in the characteristics of the patients analysed in these studies compared to those enrolled in the RCTs may explain the conflicting results. However, because the retrospective design makes it difficult to completely eliminate factors that can affect outcomes, there might be other reasons for the discrepancies, such as effect modifications with other therapies. In a 22-patient, single-centre study in 2013, Yamato et al. [39] reported the efficacy of a combination therapy with ART-123 and PMX-HP for patients with septic shock accompanied by DIC. However, the study did not evaluate the effect modification between these therapies, because, generally, a large sample size is needed to reveal a significant effect modification. The above dataset, named the Japan Septic Disseminated Intravascular Coagulation (J-Septic DIC) registry, is a unique published dataset that includes many patients who received anticoagulant therapies for septic coagulopathy and/or blood purification for septic shock [40]. The knowledge of effect modifications between these therapies, which cannot be assessed within a single RCT including only one of them, would be useful for further research and clinical decisions. Therefore, we hypothesised that there would be a significant effect modification between ART-123 and PMX-HP, which would affect survival outcomes in this dataset. In the present study, we evaluated the effect modification between the administration of ART-123 and treatment with PMX-HP on survival outcome using the dataset of the J-Septic DIC registry.

## Methods

### Study design, setting, and population

This study was conducted as a post hoc analysis of a retrospective cohort dataset of consecutive adult patients who were admitted to 42 intensive care units (ICUs) in 40 institutions throughout Japan for treatment of sepsis between January 2011 and December 2013 (the J-Septic DIC registry) [40]. We evaluated the effect modification between the administration of ART-123 and treatment with PMX-HP on survival outcome in the nationwide registry. The primary outcome was hospital mortality at discharge.

**Fig. 1:**
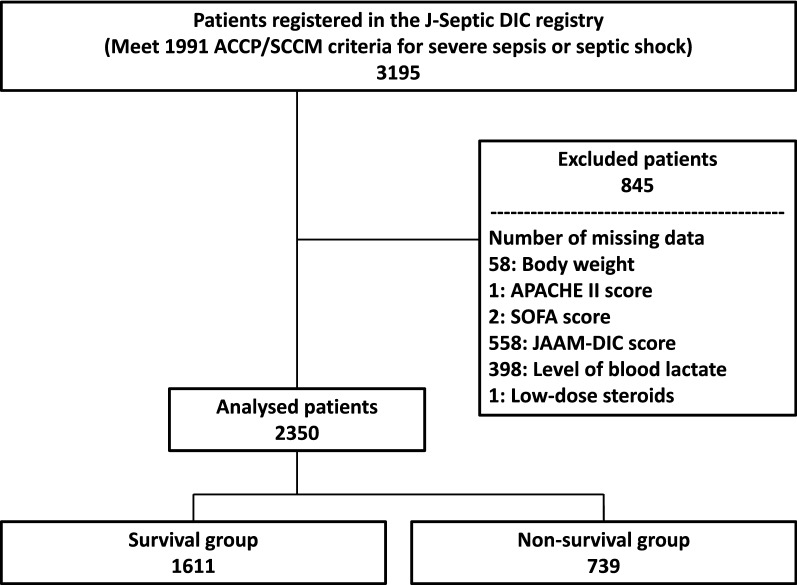
Study flowchart. The numbers of patients are indicated in each box. *ACCP* American College of Chest Physicians, *APACHE* Acute Physiology and Chronic Health Evaluation, *DIC* disseminated intravascular coagulation, *JAAM* Japanese Association for Acute Medicine, *J-Septic DIC* Japan Septic Disseminated Intravascular Coagulation, *SCCM* Society of Critical Care Medicine, *SOFA* Sequential Organ Failure Assessment

Sepsis manifestations in the registry were defined as “severe sepsis” and “septic shock” based on the conventional criteria proposed by the American College of Chest Physicians/Society of Critical Care Medicine consensus conference in 1991 [41]. Patients who were 18 years of age or older and had severe sepsis or septic shock at ICU admission were enrolled in the registry. In the present study, we excluded patients who had missing data in analysed variables, such as body weight, severity scores at ICU admission, blood lactate level on day 1, and data related to treatment (Fig. 1). Acute Physiology and Chronic Health Evaluation (APACHE) II, Sequential Organ Failure Assessment (SOFA), systemic inflammatory response syndrome (SIRS), and Japanese Association for Acute Medicine (JAAM)-DIC scores [42, 43] were used to measure severity. The JAAM-DIC score was calculated from the SIRS score, platelet count, prothrombin time-international normalised ratio, and level of fibrin/fibrinogen degradation product or D-dimer on day 1. The JAAM-DIC score was considered missing if the patient had no data for any variables used in the score calculation.

### Analysed data

We analysed the following variables collected in the J-Septic DIC registry as indicated in Table 1: patient characteristics, including ICU characteristics, severity score on day 1, blood lactate level on day 1, blood culture results, and primary infection site; therapeutic variables, including specific treatments, administration of anticoagulant for DIC treatment and anti-thrombotic drugs to treat conditions other than DIC during the first 7 days after ICU admission, and blood purifications during the first 7 days after ICU admission. Analysed outcome variables included bleeding complications (bleeding requiring transfusion, intracranial haemorrhage, bleeding requiring therapeutic intervention, and bleeding to death), days from ICU admission to hospital discharge, and hospital mortality at discharge. Age, body weight, severity scores, blood lactate levels, ventilator days, and days from ICU admission to hospital discharge were analysed as numerical variables, whereas other parameters were analysed as categorical variables.

**Table 1:** Patient characteristics, therapies, and outcomes in the survival and nonsurvival groups

	Survival (*n *= 1611)	Nonsurvival (*n* = 739)	*P* value
ICU management policy			0.034
Closed, *n* (%)	939 (58.3)	459 (62.1)	
Open, *n* (%)	386 (24.0)	180 (24.4)	
Other, *n* (%)	286 (17.8)	100 (13.5)	
Admission route to the ICU			< 0.001
Emergency department, *n* (%)	700 (43.5)	299 (40.5)	
Other hospital, *n* (%)	517 (32.1)	167 (22.6)	
Ward, *n* (%)	394 (24.5)	273 (36.9)	
Age (years)	71 (60, 79)	73 (64, 80)	< 0.001
Male sex, *n* (%)	947 (58.8)	469 (63.5)	0.031
Body weight (kg)	55.7 (47.8, 65.0)	54.2 (47.0, 63.0)	0.008
Pre-existing organ insufficiency or immunosuppression based on APACHE II score			
Liver, *n* (%)	48 (3.0)	61 (8.3)	< 0.001
Respiratory, *n* (%)	54 (3.4)	40 (5.4)	0.018
Cardiovascular, *n* (%)	78 (4.8)	67 (9.1)	< 0.001
Renal, *n* (%)	95 (5.9)	86 (11.6)	< 0.001
Immunocompromised, *n* (%)	202 (12.5)	170 (23.0)	< 0.001
Pre-existing haemostatic disorders			
Cirrhosis, *n* (%)	48 (3.0)	55 (7.4)	< 0.001
Haematological malignancy, *n* (%)	31 (1.9)	48 (6.5)	< 0.001
Chemotherapy, *n* (%)	48 (3.0)	61 (8.3)	< 0.001
Warfarin intake, *n* (%)	71 (4.4)	30 (4.1)	0.700
Other, *n* (%)	23 (1.4)	26 (3.5)	0.001
APACHE II score	21 (16, 26)	28 (21, 35)	< 0.001
SOFA score	9 (6, 11)	12 (9, 15)	< 0.001
SIRS score	3 (2, 4)	3 (2, 4)	0.031
JAAM-DIC score	3 (2, 5)	5 (3, 6)	< 0.001
Blood lactate (mmol/L)	2.6 (1.6, 4.6)	4.5 (2.1, 8.9)	< 0.001
Blood culture			< 0.001
Not taken, *n* (%)	87 (5.4)	23 (3.1)	
Positive, *n* (%)	659 (40.9)	364 (49.3)	
Negative, *n* (%)	865 (53.7)	352 (47.6)	
Microorganisms			0.033
Unknown, *n* (%)	352 (21.8)	150 (20.3)	
Virus, *n* (%)	14 (0.9)	7 (0.9)	
Gram-negative rod, *n* (%)	606 (37.6)	239 (32.3)	
Gram-positive coccus, *n* (%)	381 (23.6)	185 (25.0)	
Fungus, *n* (%)	25 (1.6)	16 (2.2)	
Mixed infection, *n* (%)	203 (12.6)	127 (17.2)	
Others, *n* (%)	30 (1.9)	15 (2.0)	
Primary source of infection			< 0.001
Unknown, *n* (%)	75 (4.7)	69 (9.3)	
Catheter-related bloodstream infection, *n* (%)	17 (1.1)	12 (1.6)	
Bone or soft tissue, *n* (%)	220 (13.7)	80 (10.8)	
Cardiovascular system, *n* (%)	33 (2.0)	12 (1.6)	
Central nervous system, *n* (%)	34 (2.1)	18 (2.4)	
Urinary tract, *n* (%)	295 (18.3)	63 (8.5)	
Lung or thoracic cavity, *n* (%)	366 (22.7)	249 (33.7)	
Abdomen, *n* (%)	541 (33.6)	228 (30.9)	
Other, *n* (%)	30 (1.9)	8 (1.1)	
Specific treatments			
Surgical intervention, *n* (%)	740 (45.9)	250 (33.8)	< 0.001
Mechanical ventilator, (days)	4 (0, 9)	5 (2, 16)	< 0.001
Vasopressor, *n* (%)	1166 (72.4)	663 (89.7)	< 0.001
Immunoglobulins, *n* (%)	520 (32.3)	271 (36.7)	0.036
Low-dose steroids, *n* (%)	330 (20.5)	286 (38.7)	< 0.001
Veno-arterial ECMO, *n* (%)	5 (0.3)	18 (2.4)	< 0.001
Veno-venous ECMO, *n* (%)	15 (0.9)	19 (2.6)	0.002
Intra-aortic balloon pumping, *n* (%)	4 (0.2)	6 (0.8)	0.081
Therapeutic interventions for DIC			
ART-123, *n* (%)	489 (30.4)	231 (31.3)	0.659
Antithrombin, *n* (%)	541 (33.6)	279 (37.8)	0.049
Protease inhibitors, *n* (%)	185 (11.5)	120 (16.2)	0.001
Heparinoids, *n* (%)	85 (5.3)	36 (4.9)	0.680
Antithrombotic drugs for conditions other than DIC			
Heparin, *n* (%)	210 (13.0)	87 (11.8)	0.392
Warfarin, *n* (%)	23 (1.4)	4 (0.5)	0.061
Anti-platelet drugs, *n* (%)	35 (2.2)	13 (1.8)	0.511
Other, *n* (%)	12 (0.7)	3 (0.4)	0.415
Nafamostat mesylate for blood purifications, *n* (%)	398 (24.7)	298 (40.3)	< 0.001
Blood purifications			
PMX-HP, *n* (%)	332 (20.6)	189 (25.6)	0.007
RRT, *n* (%)	369 (22.9)	327 (44.2)	< 0.001
RRT for non-renal indications, *n* (%)	115 (7.1)	80 (10.8)	0.003
Plasma exchange, *n* (%)	8 (0.5)	15 (2.0)	< 0.001
Concomitant treatment with ART-123 and PMX-HP, *n* (%)	164 (10.2)	83 (11.2)	0.440
Bleeding complications, *n* (%)	155 (9.6)	129 (17.5)	< 0.001
Time from ICU admission to hospital discharge (days)	33 (18, 61)	14 (3, 30.5)	< 0.001

Data are presented as *n* (%) or median (interquartile range)

*APACHE* acute physiology and chronic health evaluation, *DIC* disseminated intravascular coagulation, *ECMO* extracorporeal membrane oxygenation, *ICU* intensive care unit, *JAAM* Japanese Association for Acute Medicine, *PMX-HP* polymyxin B‑immobilised haemoperfusion, *RRT* renal replacement therapy, *SIRS* systemic inflammatory response syndrome, *SOFA* sequential organ failure assessment

### Statistical analysis

The survival and nonsurvival groups were compared in terms of their patient characteristics, therapeutic variables, and outcome variables. Categorical variables were compared using the Chi squared and Fisher’s exact tests, whereas numerical variables were compared using the Mann–Whitney U test. Categorical variables were presented as numbers and percentages, whereas numerical variables were summarised using the median and interquartile range (IQR).

The significance of effect modification between the administration of ART-123 and treatment with PMX-HP was evaluated using the multivariate Cox regression model until day 90. The product term was inputted into the Cox regression model for hospital mortality adjustment with most analysed patient characteristics and therapeutic variables as covariates. The presence of haemostatic disorders caused by liver cirrhosis was excluded as a variable because of the concerns about collinearity with the presence of chronic liver failure. In addition, the therapeutic variables veno-arterial and veno-venous extracorporeal membrane oxygenation, intra-aortic balloon pumping, as well as warfarin and other drug use for conditions other than DIC were excluded from the Cox regression model, because log–log plots of these variables revealed unsatisfied proportional hazard assumption of these variables. Furthermore, a subgroup analysis of patients who required vasopressors was performed using a similar Cox regression model to evaluate the significance of the effect modification between the administration of ART-123 and treatment with PMX-HP in shock-suspected patients.

We did not impute any missing data and performed a complete case analysis for all analyses. All statistical analyses were performed using IBM SPSS Statistics version 26 (IBM Co., Armonk, New York, USA) and differences were considered statistically significant if *P* < 0.05.

## Results

### Patient characteristics in the survival and nonsurvival groups

Of the 3195 patients in the J-Septic DIC registry, 2350 patients were included in the final analysis after the exclusion of 845 patients that missed data for any of the analysed variables (Fig. 1). The median patient age was 71 years (IQR: 62, 80 years) and 60.3% (1416/2350) of the patients were male. On ICU admission day, the median APACHE II, SOFA, SIRS, and JAAM-DIC scores were 23 (IQR: 17, 29), 10 (IQR: 7, 13), 3 (IQR: 2, 4), and 4 (IQR: 2, 6), respectively. The rate of hospital mortality was 31.4% (739/2350).

Table 1 lists patient characteristics, therapeutic variables, and outcome variables of the survival and nonsurvival groups. Patient age and severity scores were significantly higher in the nonsurvival group, and bleeding complications were more frequently observed (17.5% vs. 9.6%; *P* < 0.001). Among the variables evaluated for effect modification, PMX-HP treatment was more frequent in the nonsurvival group (25.6% vs. 20.6%; *P* = 0.007), whereas the proportions of patients that received ART-123 were not significantly different between the two groups (31.3% vs. 30.4%; *P* = 0.659).

### Effect modification of combined ART-123/PMX-HP treatment on survival outcome

Table 2 shows the covariate-adjusted Cox regression model. Prior to the introduction of the product term, the administration of ART-123 was independently associated with the survival outcome (adjusted hazard ratio [HR]: 0.834, 95% confidence interval [CI] 0.695–0.999; *P* = 0.049). Table 3 shows the adjusted HR, 95% CI, and *P* values of the product term between ART-123 and PMX-HP, and related therapeutic variables after the product term was introduced into the Cox regression model. The effect modification between the administration of ART-123 and PMX-HP treatment significantly affected the survival outcome (adjusted HR: 0.667, 95% CI 0.462–0.961; *P* = 0.030) (Table 3a).

**Table 2:** Cox regression model adjusted for patient characteristics and therapeutic variables for hospital mortality

	Adjusted hazard ratio	95% confidence interval	*P* value
ICU management policy			
Closed	Reference		
Open	1.113	0.919–1.348	0.275
Other	0.744	0.584–0.947	0.017
Admission route to the ICU			
Emergency department	Reference		
Another hospital	0.877	0.713–1.078	0.211
Ward	0.981	0.812–1.187	0.847
Age (years)	1.013	1.006–1.020	< 0.001
Male sex	1.125	0.947–1.337	0.179
Body weight (kg)	0.991	0.984–0.997	0.004
Pre-existing organ insufficiency or immunosuppression based on APACHE II score			
Liver	1.278	0.952–1.716	0.102
Respiratory	1.422	1.020–1.983	0.038
Cardiovascular	1.354	1.024–1.790	0.034
Renal	1.460	1.132–1.885	0.004
Immunocompromised	1.081	0.864–1.352	0.497
Pre-existing haemostatic disorders			
Haematological malignancy	1.118	0.771–1.619	0.556
Chemotherapy	0.982	0.710–1.360	0.915
Warfarin intake	0.776	0.524–1.150	0.206
Other	1.464	0.939–2.283	0.093
APACHE II score	1.035	1.023–1.047	< 0.001
SOFA score	1.088	1.054–1.123	< 0.001
SIRS score	0.953	0.869–1.047	0.316
JAAM-DIC score	1.032	0.987–1.079	0.161
Blood lactate (mmol/L)	1.083	1.066–1.100	< 0.001
Blood culture			
Not taken	Reference		
Positive	1.083	0.690–1.700	0.728
Negative	0.885	0.573–1.365	0.581
Microorganisms			
Unknown	Reference		
Virus	0.945	0.400–2.231	0.898
Gram-negative rod	0.791	0.612–1.023	0.074
Gram-positive coccus	0.902	0.685–1.188	0.463
Fungus	1.158	0.644–2.083	0.625
Mixed infection	1.034	0.784–1.364	0.810
Others	1.126	0.642–1.975	0.678
Primary source of infection			
Unknown	Reference		
Catheter-related bloodstream infection	0.631	0.317–1.254	0.189
Bone or soft tissue	0.765	0.525–1.116	0.165
Cardiovascular system	0.566	0.289–1.109	0.097
Central nervous system	0.579	0.328–1.023	0.060
Urinary tract	0.538	0.364–0.794	0.002
Lung or thoracic cavity	1.084	0.803–1.464	0.598
Abdomen	0.774	0.555–1.079	0.131
Other	0.625	0.290–1.348	0.231
Specific treatments			
Surgical intervention	0.766	0.619–0.948	0.014
Mechanical ventilator (days)	0.977	0.967–0.987	< 0.001
Vasopressor	1.290	0.979–1.701	0.070
Immunoglobulins	0.859	0.719–1.027	0.095
Low-dose steroids	1.420	1.190–1.695	< 0.001
Therapeutic interventions for DIC			
ART-123	0.834	0.695–0.999	0.049
Antithrombin	0.875	0.728–1.053	0.158
Protease inhibitors	0.916	0.727–1.152	0.452
Heparinoids	1.014	0.706–1.457	0.940
Anti-thrombotic drugs for conditions other than DIC			
Heparin	0.690	0.537–0.886	0.004
Anti-platelet drugs	0.646	0.342–1.221	0.179
Nafamostat mesylate for blood purifications	0.702	0.553–0.889	0.003
Blood purifications			
PMX-HP	0.897	0.720–1.118	0.333
RRT	1.383	1.090–1.756	0.008
RRT for non-renal indications	1.300	0.992–1.704	0.057
Plasma exchange	1.498	0.838–2.677	0.172

*APACHE* acute physiology and chronic health evaluation, *DIC* disseminated intravascular coagulation, *ECMO* extracorporeal membrane oxygenation, *ICU* intensive care unit, *JAAM* Japanese Association for Acute Medicine, *PMX-HP* polymyxin B‑immobilised haemoperfusion, *RRT* renal replacement therapy, *SIRS* systemic inflammatory response syndrome, *SOFA* sequential organ failure assessment

**Table 3:** Adjusted hazard ratios of product terms between ART-123 and PMX-HP and related therapeutic variables

	Adjusted hazard ratio	95% confidence interval	*P* value
(a) Overall			
ART-123 × PMX-HP	0.667	0.462–0.961	0.030
ART-123	0.774	0.639–0.937	0.009
PMX-HP	0.872	0.699–1.086	0.222
(b) Patients who required vasopressors			
ART-123 × PMX-HP	0.637	0.439–0.925	0.018
ART-123	0.790	0.649–0.960	0.018
PMX-HP	0.889	0.710–1.112	0.301

*PMX-HP* polymyxin B‑immobilised haemoperfusion

The adjusted hazard ratio, 95% confidence interval, and *P* values indicate those calculated after the product term was introduced into the Cox regression model shown in Table 2 or 4

Table 4 shows the covariate-adjusted Cox regression model for the subgroup of 1829 patients who required vasopressors. The effect modification between ART-123 administration and PMX-HP treatment significantly affected the survival outcome in the subgroup (adjusted HR: 0.637, 95% CI 0.439–0.925; *P* = 0.018) (Table 3b).

**Table 4:** Cox regression model adjusted for patient characteristics and therapeutic variables to assess hospital mortality in patients who received vasopressors

	Adjusted hazard ratio	95% confidence interval	*P* value
ICU management policy			
Closed	Reference		
Open	1.076	0.876–1.320	0.486
Other	0.746	0.579–0.962	0.024
Admission route to the ICU			
Emergency department	Reference		
Another hospital	0.858	0.689–1.069	0.172
Ward	0.965	0.791–1.178	0.727
Age (years)	1.014	1.007–1.022	< 0.001
Male sex	1.219	1.015–1.464	0.034
Body weight (kg)	0.992	0.986–0.999	0.027
Pre-existing organ insufficiency or immunosuppression based on APACHE II score			
Liver	1.210	0.888–1.649	0.228
Respiratory	1.412	0.997–1.998	0.052
Cardiovascular	1.413	1.061–1.883	0.018
Renal	1.467	1.120–1.922	0.005
Immunocompromised	1.088	0.861–1.376	0.479
Pre-existing haemostatic disorders			
Haematological malignancy	1.070	0.720–1.591	0.737
Chemotherapy	0.981	0.699–1.377	0.912
Warfarin intake	0.807	0.540–1.207	0.296
Other	1.202	0.716–2.019	0.486
APACHE II score	1.037	1.025–1.051	< 0.001
SOFA score	1.078	1.043–1.115	< 0.001
SIRS score	0.958	0.868–1.058	0.400
JAAM-DIC score	1.029	0.982–1.079	0.226
Blood lactate (mmol/L)	1.083	1.065–1.101	< 0.001
Blood culture			
Not taken	Reference		
Positive	1.022	0.623–1.678	0.930
Negative	0.813	0.504–1.313	0.398
Microorganisms			
Unknown	Reference		
Virus	0.692	0.245–1.956	0.488
Gram-negative rod	0.800	0.607–1.052	0.111
Gram-positive coccus	0.939	0.699–1.262	0.678
Fungus	1.245	0.683–2.269	0.474
Mixed infection	1.031	0.764–1.390	0.843
Others	1.061	0.566–1.990	0.853
Primary source of infection			
Unknown	Reference		
Catheter-related bloodstream infection	0.653	0.325–1.312	0.232
Bone or soft tissue	0.816	0.547–1.218	0.320
Cardiovascular system	0.577	0.284–1.171	0.128
Central nervous system	0.708	0.379–1.323	0.279
Urinary tract	0.544	0.357–0.830	0.005
Lung or thoracic cavity	1.114	0.807–1.537	0.512
Abdomen	0.784	0.551–1.116	0.176
Other	0.632	0.277–1.439	0.274
Specific treatments			
Surgical intervention	0.767	0.614–0.958	0.019
Mechanical ventilator (days)	0.974	0.964–0.985	< 0.001
Immunoglobulins	0.885	0.735–1.066	0.198
Low-dose steroids	1.387	1.154–1.666	< 0.001
Therapeutic interventions for DIC			
ART-123	0.849	0.704–1.025	0.089
Antithrombin	0.885	0.730–1.072	0.210
Protease inhibitors	0.893	0.702–1.137	0.358
Heparinoids	1.045	0.709–1.542	0.823
Anti-thrombotic drugs for conditions other than DIC			
Heparin	0.696	0.536–0.904	0.007
Anti-platelet drugs	0.706	0.361–1.382	0.310
Nafamostat mesylate for blood purifications	0.699	0.547–0.893	0.004
Blood purifications			
PMX-HP	0.915	0.731–1.146	0.440
RRT	1.398	1.092–1.789	0.008
RRT for non-renal indications	1.323	1.005–1.740	0.046
Plasma exchange	1.632	0.864–3.083	0.131

*APACHE* acute physiology and chronic health evaluation, *DIC* disseminated intravascular coagulation, *ECMO* extracorporeal membrane oxygenation, *ICU* intensive care unit, *JAAM* Japanese Association for Acute Medicine, *PMX-HP* polymyxin B‑immobilised haemoperfusion, *RRT* renal replacement therapy, *SIRS* systemic inflammatory response syndrome, *SOFA* sequential organ failure assessment

## Discussion

Our results demonstrate that the main effect of the administration of ART-123 may be beneficial for survival outcome, and its effects were augmented by a significant effect modification upon co-treatment with PMX-HP. This study is the first to demonstrate a significant positive effect modification between the administration of ART-123 and treatment with PMX-HP on the survival outcome of patients with sepsis.

In 2016, Hayakawa et al. [35] used propensity score analysis to analyse J-Septic DIC registry data, and reported a significantly improved survival outcome following ART-123 treatment in patients with sepsis-induced DIC. In that report, survival times between propensity score-matched ART-123 and control groups were significantly different (HR: 0.781, 95% CI 0.624–0.977; *P* = 0.030). In this study, although the inclusion criteria and statistical model used were different, the main effect of ART-123 administration, before adjusting for product terms, was also significantly beneficial to survival (adjusted HR: 0.834, 95% CI 0.695–0.999; *P* = 0.049). However, we also observed a significant effect modification between treatments with ART-123 and PMX-HP. Thus, the effect of ART-123 observed in the study by Hayakawa et al. may also have been influenced by that effect modification. In that study, 31.6% of patients in the ART-123 group after propensity score matching also received PMX-HP therapy. In addition, in 2017, Nakamura et al. [36] reported a significant positive effect of the PMX-HP therapy on survival outcome using the same dataset and propensity score matching. In their study, they used a different indicator for survival outcome (the odds ratio for hospital mortality) in the population different from that in the present study, making the comparison of the results of these two studies complicated. However, 38.9% of patients in the PMX-HP group after propensity score matching received ART-123, thus the effect of PMX-HP observed in the study by Nakamura et al. [36] might also have been influenced by this effect modification. Propensity score matching and other propensity score analyses can be useful to control biases in observational studies [44]; however, the bias reducing capabilities of propensity scores may decrease when the propensity scores are estimated without considering interactions [45].

The mechanism of the effect modification between ART-123 and PMX-HP treatments remains unclear. Although the predominant effect of PMX-HP is thought to be endotoxin removal, it has also been reported that PMX-HP traps activated leukocytes and platelets [28]. Activated blood cells are known to mediate the development of coagulopathy, which is followed by organ dysfunction and shock during sepsis. Iba et al. [46] suggested that the adsorption of such activated blood cells might be a therapeutic strategy against the complex mechanism of shock development during sepsis, in which the coagulation pathway plays an important role. Yamato et al. [39] reported efficacy for ART-123/PMX-HP combination therapy in patients with septic shock accompanied by DIC, suggesting that simultaneous control of high-mobility group box-1 protein, a late mediator of sepsis, through ART-123 and PMX-HP therapy might be a putative mechanism underpinning the beneficial effect. Although the present study could not reveal the detailed mechanism of the effect modification between ART-123 and PMX-HP treatments, our analysis of a large multicentre sample of 2350 patients supports the possibility of clinical efficacy of the combination reported by Yamato et al. We believe that in addition to the independent primary mechanisms of each therapy (anticoagulation and endotoxin removal), the simultaneous targeting of multiple mediators related to the development of organ dysfunction and shock in sepsis likely explains the effect modification between these therapies.

The use of a Japanese nationwide dataset, which included patients that received several novel interventions for sepsis, was a particular strength of the present study. Analysis of effect modifications is difficult to perform with a small sample size dataset, because the sample size of each variable evaluated for the effect modifications is smaller than overall sample size. In this study, ART-123 was administered to 720 patients (30.6% of the total cohort), PMX-HP was performed in 521 patients (22.2% of the total cohort), and concomitant therapy was administered to 247 patients (10.5% of the total cohort, 34.3% of patients who received ART-123, and 47.4% of the patients who received PMX-HP). In Japan, ART-123 was approved for the indication of DIC by the Ministry of Health, Labour and Welfare in 2008 [47], whereas PMX-HP treatment was approved for the indication of severe Gram-negative bacterial infection in 1994 [27]. To the best of our knowledge, it is only in Japan that both these therapies can be used in general clinical setting. Thus, the nationwide dataset compiled in Japan, which comprised hundreds of patients who received unique treatments for sepsis, was useful for the evaluation of effect modification between these relatively novel approaches. It should be noted that numerous RCTs designed to evaluate the effects of each individual therapy in comparison to the standard of care [20–22, 25, 31–33] could not examine effect modifications between the novel treatments.

This study has several limitations. First, its retrospective observational design is associated with a risk of unmeasured or unknown biases. Second, approximately a quarter of eligible patients were excluded because of missing data for some of the analysed variables. This selection process might also have introduced the risk of bias. However, there were several analysed variables that were not reported in over 10% of patients; therefore, we did not use the multiple imputation method. Third, the J-Septic DIC dataset is relatively old, and the definitions of severe sepsis and septic shock used in the dataset were proposed in 1991 [41], whereas the current definitions of sepsis and septic shock were published in 2016 [5], after the J-Septic DIC registry was compiled. Fourth, we used the JAAM-DIC score, which is predominantly used in Japan, and our conclusions might not extend to hospitals that use the International Society of Thrombosis and Haemostasis (ISTH) criteria [48]. However, we found that the JAAM-DIC score diagnosed most of the overt DIC cases, as do the ISTH criteria [42, 43, 49, 50]. In addition, 911 patients (28.5%) had missing data in variables necessary for the calculation of the ISTH criteria. Therefore, we reasoned that it would be difficult to use the ISTH score in the present study, as it would require adjustments to many factors in the Cox regression model. Fifth, in Japan, continuous intracircuit infusion of nafamostat mesylate (NM) may be used as an anticoagulant treatment during PMX-HP. Because the main effect of NM was significant in the Cox regression model used in the present study, there is a possibility that the results were affected by NM infusion during PMX-HP. However, no significant effect modification between ART-123 and NM treatments was observed (data not shown). Sixth, we did not evaluate simple main effects of ART-123 and PMX-HP, because the subgroups did not have adequate sample size to be evaluated by the Cox regression model used in the present study. Further studies will be needed to validate our findings; however, the results of the present study might help designing optimal RCTs to evaluate the effects of ART-123 and/or PMX-HP and impact clinical decision-making.

## Conclusion

A significant beneficial effect modification on survival outcome between the administration of ART-123 and PMX-HP treatment was observed in patients with sepsis. Further study is needed to evaluate the effects of combination therapy with ART-123 and PMX-HP on survival outcomes.

## Data Availability

The dataset analysed during the current study is available in Hayakawa M., et al. Nationwide registry of sepsis patients in Japan focused on disseminated intravascular coagulation 2011–2013, *Scientific Data.* 2018;5:180243. 10.1038/sdata.2018.243.

## Abbreviations

APACHE: Acute Physiology and Chronic Health Evaluation
CI: Confidence interval
DIC: Disseminated intravascular coagulation
HR: Hazard ratio
ICU: Intensive care unit
IQR: Interquartile range
JAAM: Japanese Association for Acute Medicine
J-Septic DIC: Japan Septic Disseminated Intravascular Coagulation
PMX-HP: Polymyxin B‑immobilised haemoperfusion
RCT: Randomised controlled trial
RRT: Renal replacement therapy
SIRS: Systemic inflammatory response syndrome
SOFA: Sequential Organ Failure Assessment
